# Effects of vaping on kidney function: a systematic review on acute kidney injury and chronic kidney disease

**DOI:** 10.1590/2175-8239-JBN-2024-0216en

**Published:** 2025-10-13

**Authors:** Guilherme Nobre Nogueira, Elizabeth De Francesco Daher

**Affiliations:** 1 Universidade Federal do Ceará, Faculdade de Medicina, Departamento de Medicina Interna, Fortaleza, CE, Brazil.; 2 Universidade Federal do Ceará, Faculdade de Medicina, Departamento de Medicina Interna, Programa de Pós-Graduação em Ciências Médicas, Fortaleza, CE, Brazil.

**Keywords:** Vaping, Nicotine, Acute Kidney Injury, Renal Insufficiency, Chronic, Microangiopathy, Glomerular Damage, Nephritis, Interstitial, Glomerulosclerosis, Focal Segmental

## Abstract

The increase use of vaping, especially in the younger population, has led to increased scrutiny of its health effects, particularly on renal function. This article reviews the current literature on the association between vaping, nicotine exposure, and renal impairment, focusing on the development of acute kidney injury (AKI) and chronic kidney disease (CKD). Nicotine and other chemicals in e-liquids may induce microangiopathy, leading to vasoconstriction and reduced renal perfusion, thus contributing to AKI. Chronic exposure to nicotine also promotes an inflammatory response, increasing the risk of interstitial nephritis. Additionally, glomerular damage due to continuous use of vapers has been linked to albuminuria and progression of focal segmental glomerulosclerosis (FSGS), a condition that can lead to CKD. This article highlights the need for further research to clarify the kidney risks associated with vaping and provides information for public health policies on nicotine use.

## Introduction

The increase use of vapers (electronic cigarettes) has sparked a growing interest in their potential health effects. Initially marketed as a safer alternative to conventional cigarettes, vaping has gained widespread popularity, especially among the younger population. While the effects of vaping on respiratory and cardiovascular systems have been widely researched, the effects on other organ systems, particularly the kidneys, remain underexplored. The kidneys filter waste products from the bloodstream, maintain electrolyte balance, and regulate blood pressure. Consequently, any detrimental effects on kidney function could have severe and long-lasting health implications^1,2^.

Given the chemical composition of the liquids used in vapers such as nicotine and other potentially harmful substances, the effects on renal function are a growing concern. Some studies suggest that nicotine can induce vasoconstriction in the kidneys, contributing to acute kidney injury (AKI) and chronic kidney disease (CKD). In light of the potential long-term renal damage, this systematic review and meta-analysis aims to investigate the association between vaping and renal function, focusing on the risks of AKI and CKD^3,4^.

The goal of this article is to provide a comprehensive analysis of the existing literature regarding the potential impacts of vaping on renal function. Specifically, this study seeks to compare the effects of vaping with those of conventional tobacco smoking and the non-use of nicotine products. By identifying any differences related to different types of vapers and nicotine concentrations, this analysis could have significant implications for public health policies and recommendations for nicotine use.

## Method

This systematic review followed the PICOT framework. The population of interest (P) were individuals with no pre-existing kidney impairments. The intervention (I) was the use of vapers (e-cigarettes) containing various nicotine concentrations. The comparator (C) consisted of conventional cigarette users and non-users of nicotine products. The outcomes (O) were changes in renal function, including the development of AKI and CKD, as well as measurements of serum creatinine levels and glomerular filtration rate (GFR). Studies had to have a minimum follow-up period of six months to be included.

The article aimed to answer the following question: What is the association between vaping (e-cigarettes) and AKI and CKD in adults, compared to conventional tobacco users and non-nicotine users, in studies with a minimum follow-up of 6 months?

The search strategy employed relevant terms across PubMed, Embase, and Cochrane Library databases [(“vapers” OR “e-cigarettes” OR “electronic cigarettes” OR “e-cig” OR “vaping” OR “vape” OR “vaporizer” OR “vapor products”) AND (“renal impairment” OR “kidney function” OR “renal function” OR “acute kidney injury” OR “AKI” OR “chronic kidney disease” OR “CKD” OR “glomerular filtration rate” OR “GFR” OR “creatinine” OR “renal failure” OR “kidney damage” OR “nephropathy”) AND (“tobacco” OR “smoking” OR “nicotine” OR “non-smokers” OR “control group” OR “cigarette” OR “smoking cessation” OR “tobacco smoke” OR “tobacco use” OR “nicotine addiction” OR “smoking habits” OR “smoking-related diseases”)].

**Figure 1:**
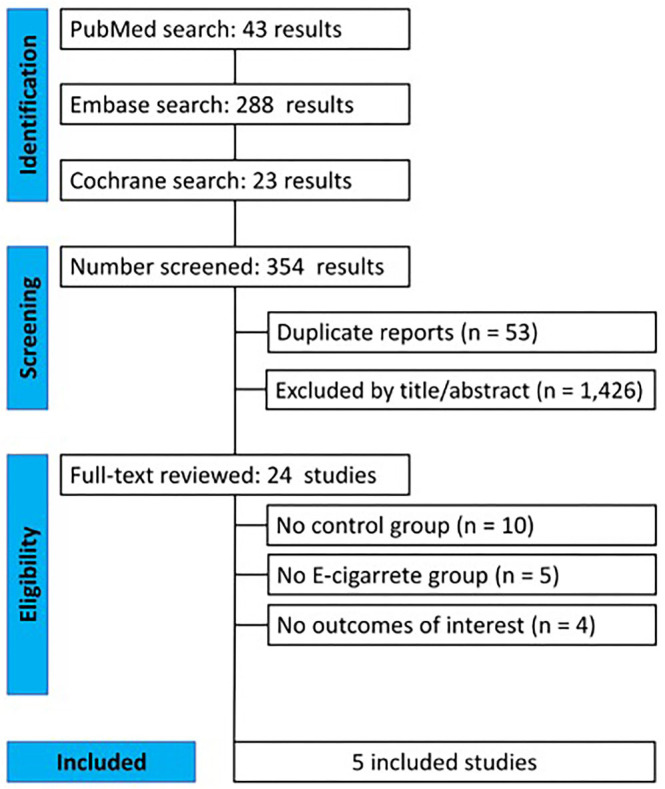
PRISMA flow diagram of study screening and selection.

A total of 354 articles were initially identified and after removing 53 duplicates, 301 were screened based on titles and abstracts. Twenty-four full-text articles were reviewed, 10 did not mention a control group, 5 did not have the use of e-cigarette as an intervention group, and 4 did show outcomes of interest. Finally, five articles met the inclusion criteria for this analysis, as cited in Figure 1.

## Results

The included studies (Table 1) consistently reported an association between vaping and impaired kidney function. Users of e-cigarettes showed elevated serum creatinine levels and a decline in GFR compared to non-users of nicotine products. Furthermore, the risk of AKI and the progression of CKD were significantly higher among vapers compared to non-smokers, though the effects were somewhat comparable to those observed in conventional cigarette smokers.

**Table 1:** Description of the incluDeD articles

Study	N	Intervention	Control group	Study design	Vaping Time	Follow-up	Female sex	Mean age
Poudel 2024	270	Tobacco smokers and e-cigarette users	Non-smokers	Observational	3.5 ± 2.2 years	Not specified	174/270	21.2 ± 2.3
Lyytinen 2023	22	30 puffs of EC aerosol with nicotine	Non-smokers	Randomized	Not specified	1 week washout period	15/22	27 ± 7
Sakamaki-Ching 2020	53	E-cigarette and cigarette smoking	Non-smokers	Cross-sectional	Not specified	Not specified	33/53	Not specified
Bragina 2019	270	Tobacco and e-cigarette use	Non-smokers	Observational	3.5 ± 2.2 years	Not specified	174/270	21.2 ± 2.3
Daisy 2024	1315	Exclusive nicotine and non-nicotine e-cigarette use	Non-smokers	Cross-sectional	Not specified	Not specified	49.3%	14.9 (median)

Among the five included studies^5,6,7,8,9^, one large cohort study (Daisy, 2024) reported that individuals who used vapers with high nicotine concentrations exhibited a notably higher risk of kidney dysfunction compared to those using lower concentrations. Another study highlighted that prolonged vaping might exacerbate kidney impairment due to continuous exposure to harmful chemicals found in e-liquids, including heavy metals and other toxic compounds.

In Daisy, 2024, nicotine e-cigarette users had higher plasma levels of cotinine (61.3 ng/mg creatinine) and 3-HC (118.1 ng/mg creatinine) compared to non-users (cotinine 0.4 ng/mg creatinine, 3-HC 0.8 ng/mg creatinine), indicating greater nicotine exposure and potential harm^5^.

Lyytinen, 2023, demonstrated that e-cigarette aerosol with nicotine increased platelet thrombus formation and systolic blood pressure by +9.25 mmHg. Conversely, the e-cigarette aerosol without nicotine did not show significant effects on thrombogenesis or microvascular function, indicating the harmful effects associated with nicotine exposure^6^.

Sakamaki-Ching, 2020, found that e-cigarette users had elevated oxidative stress markers, including 8-OHdG (442.8 ± 300.7 ng/mg) and 8-Isoprostane (750.8 ± 433 pg/mg), compared to non-smokers. Additionally, their levels of metallothionein and zinc were significantly higher, suggesting increased metal exposure linked to oxidative DNA damage^7^.

In Poudel 2024, e-cigarette users exhibited significantly higher levels of albuminuria (252.7 ± 209.1 mg/day) compared to tobacco smokers (106.0 ± 76.5 mg/day) and non-smokers (29.8 ± 31.2 mg/day), suggesting greater renal dysfunction. Similarly, their vascular augmentation index (Alx) was elevated (–6.9 ± 11.4%) relative to tobacco smokers (–12.9 ± 13.7%) and non-smokers (–22.0 ± 10.6%)^8^.

Bragina, 2019, had similar findings as Poudel, with e-cigarette users showing greater albuminuria and vascular dysfunction than tobacco smokers and non-smokers, consistent across both studies^9^.

Poudel, 2024, and Bragina, 2019, both measured albuminuria and vascular augmentation index (Alx). In both studies, e-cigarette users showed the highest levels of albuminuria and Alx, indicating worse kidney and vascular dysfunction compared to both tobacco smokers and non-smokers. Tobacco smokers had intermediate results, with elevated albuminuria and Alx compared to non-smokers but lower than e-cigarette users. Non-smokers had the lowest levels in both outcomes^8,9^.

Lyytinen, 2023, and Sakamaki-Ching, 2020, observed increased markers of potential harm (oxidative stress, platelet thrombus formation) in e-cigarette users. Lyytinen 2023 found that nicotine-containing e-cigarette aerosol with increased platelet thrombus formation and impaired microvascular dilation compared to nicotine-free aerosol. Sakamaki-Ching, 2020, identified increased oxidative stress markers (8-OHdG and 8-Isoprostane) in e-cigarette users, correlating with higher metal exposure (especially zinc) and oxidative DNA damage. In both studies, the nicotine in e-cigarettes was a key driver of adverse outcomes such as increased thrombogenesis and oxidative stress^6,7^. Nicotine e-cigarette users in both studies had significantly elevated levels of cotinine, a marker of nicotine exposure.

Daisy, 2024, showed a dose-response relationship between vaping frequency and cotinine/3-HC levels, with higher levels in frequent users. Sakamaki-Ching, 2020, found a positive correlation between cotinine levels and metal concentrations, which was linked to oxidative damage, reinforcing the link between e-cigarette use and kidney injury^5,7^.

Both Sakamaki-Ching, 2020, and Lyytinen, 2023, reported outcomes related to oxidative stress: Sakamaki-Ching, 2020, identified elevated oxidative stress markers like 8-OHdG in e-cigarette users, linked to metal exposure. Lyytinen, 2023, highlighted increased oxidative damage through impaired microvascular function following nicotine exposure^6,7^.

All studies observed elevated markers (e.g., albuminuria, cotinine, oxidative stress, or thrombogenicity) in e-cigarette users compared to non-users. In all studies, nicotine was identified as a key factor contributing to negative outcomes, including increased oxidative stress, impaired vascular function, and increased thrombus formation^5,9^.

**Figure 2:**
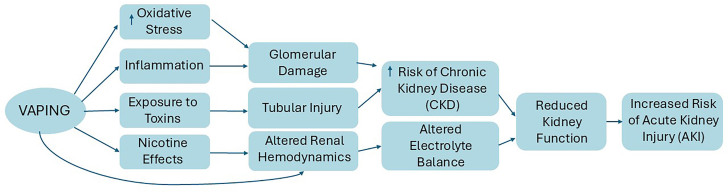
Potential effects and mechanisms of vaping on kidney health.

These similarities among the studies underscore the adverse effects of e-cigarette use, particularly when nicotine is present, on vascular health, oxidative stress, and kidney function, which are represented in the Figure 2.

## Discussion

Recent studies have confirmed that nicotine, regardless of the delivery method, poses a significant risk to kidney health. For instance, Patel et al.^10^ found that nicotine exposure causes vasoconstriction and oxidative stress in kidney tissues, increasing the likelihood of AKI and CKD in chronic users. These findings align with the results of this review, indicating that vapers are at comparable risk of kidney damage to conventional smokers.

In addition to nicotine, several compounds found in the vapor produced by e-cigarettes, such as propylene glycol, glycerol, and flavoring agents, may contribute to renal dysfunction. Zhang et al.^11^ explored how the breakdown products of these compounds might lead to endothelial dysfunction in renal vasculature, which could exacerbate or initiate kidney injury. These findings support the hypothesis that, while vaping may reduce certain risks associated with traditional cigarettes, it introduces new risks to kidney function.

In contrast, some studies suggest that the type of vaping device and the concentration of nicotine may modify the risks to kidney health. For example, individuals using low-nicotine vapers or those designed to deliver nicotine more slowly appeared to have a reduced risk of kidney impairment compared to high-nicotine vapers. Further research is needed to confirm these observations and understand the mechanisms by which different vaping products impact kidney function.

Current and former smokers have a significantly higher risk of hospitalization due to AKI compared to never-smokers. This risk decreases over time following smoking cessation, but it may persist for up to 30 years^12^. Exposure to e-cigarette vapor, especially when combined with a high-fat diet, increases oxidative stress, DNA damage, and inflammatory responses in the kidneys, accelerating the development of renal pathologies^13^. Patients with a history of AKI have an increased risk of developing CKD and end-stage kidney disease (ESKD). This risk is higher compared to those without a history of AKI, and the severity of the AKI is associated with worse outcomes^14^.

Smokers with AKI have significantly lower levels of vitamin D compared to non-smokers. This deficiency can exacerbate inflammatory complications and worsen kidney outcomes. Vitamin D is known to play a crucial role in modulating immune responses and reducing inflammation, and its deficiency may lead to increased susceptibility to kidney damage and progression of kidney disease^15^.

Studies indicate that both conventional smoking and vaping are associated with an increased risk of acute and chronic kidney injury. Conventional cigarette smokers have an elevated risk of AKI, which may persist for decades after quitting smoking. Moreover, smokers with AKI often have vitamin D deficiency, which can further exacerbate kidney outcomes.

Chronic inflammation induced by nicotine is also a significant factor in the development of interstitial nephritis. While there are not many studies specifically focusing on this condition in vape users, existing literature on traditional smoking suggests that nicotine-mediated kidney inflammation, exacerbated by altered immune responses, can lead to interstitial nephritis. The same process can be extrapolated to vape users, considering the similarity of damage caused by chronic nicotine exposure^4,8,10^.

A relevant aspect is the association between nicotine and glomerular dysfunction. The increased levels of albuminuria observed in vape users, as seen in the reviewed studies, are indicative of glomerular damage, which may progress to FSGS. Additionally, continuous exposure to heavy metals found in e-liquids may intensify the glomerular sclerosis process, suggesting that high-nicotine vape users may have an elevated risk of developing FSGS^6,10^.

In contrast, non-nicotine users generally exhibit better kidney health outcomes, indicating that nicotine-related oxidative and inflammatory damage is related to AKI and CKD. This highlights the need for further research to clarify the long-term renal impacts of vaping compared to traditional smoking and non-use of tobacco products.

In addition to nicotine, non-nicotine components of vaping aerosols—such as free fatty acids (FFAs) and other lipophilic agents—may play a role in kidney injury. FFAs have been shown to induce lipotoxicity in renal endothelial cells and podocytes by promoting mitochondrial dysfunction, endoplasmic reticulum stress, and reactive oxygen species (ROS) generation^16,17^. These mechanisms may disrupt glomerular integrity and accelerate CKD progression. Recent in vitro studies suggest that flavoring chemicals used in e-liquids can also increase FFA levels and alter lipid metabolism in exposed cells^18^, which could potentiate nephrotoxicity independently of nicotine. The interplay between lipid peroxidation, inflammation, and cellular stress responses may therefore provide an additional explanatory pathway for kidney damage observed in e-cigarette users.

Vaping may also affect other organs within the genitourinary tract, particularly the bladder. Recent studies have raised concerns about the carcinogenic potential of e-cigarette use, with special emphasis on urothelial malignancies. Urinary excretion of metabolites derived from nitrosamines and polycyclic aromatic hydrocarbons, compounds found in both traditional and electronic cigarettes, may result in direct exposure of the bladder epithelium to carcinogens. For example, Lee et al.^19^ identified elevated levels of urinary N-nitrosamines in e-cigarette users, which are known bladder carcinogens. Additionally, Werley et al.^20^ reported DNA damage and oxidative stress in urothelial cells exposed to e-cigarette aerosol extracts. While epidemiologic data are still limited, preclinical findings support a mechanistic link between chronic vaping and increased risk of bladder cancer, particularly in individuals with other risk factors such as male sex, occupational exposure, and genetic predisposition^19,20,21^. These emerging concerns highlight the need for prospective studies to evaluate the long-term oncologic impact of vaping on the genitourinary system.

Conventional cigarette smoking is a well-established risk factor for the development of CKD, the acceleration of pre-existing CKD, and kidney allograft failure^22,23^. Importantly, smoking cessation has been shown to effectively reduce this risk^23^. In recent years, however, there has been a dramatic increase in the use of electronic cigarettes (e-cigs), also known as vapes. Despite their growing popularity, the effects of e-cig use on kidney health compared to conventional cigarettes are unknown^22^.

Experimental animal studies suggest that components and aerosols from e-cigarettes may cause structural and functional damage to kidneys. For example, mice exposed to nicotine-containing e-cig vapor for six months demonstrated a significant reduction in glomerular filtration rate alongside increased renal fibrosis^24^. Other research in rats points to the e-cig refill liquid itself, rather than nicotine, as a primary cause of kidney structural damage^22^. Moreover, in obese and diabetic mice, long-term exposure to nicotine-containing e-cig aerosols induced significant changes in gene expression related to CKD progression. Complementing these findings, in vitro studies have revealed that vanillin, a common flavoring agent in e-cig liquids, exhibits cytotoxic effects on human renal tubular cells by impairing mitochondrial viability and autophagic pathways. Additionally, maternal exposure to e-cig vapor during pregnancy and lactation in mice resulted in offspring with reduced glomerular density and increased albuminuria^22^.

Despite these experimental findings, evidence from human studies remains limited and primarily derives from a small number of cross-sectional investigations^22^. A study involving adolescents and young adults with pediatric-onset CKD observed a trend toward increased proteinuria among e-cig users, although without statistical significance. Another cross-sectional study conducted in healthy young individuals reported a higher prevalence of albuminuria among vape users compared to traditional cigarette smokers and non-smokers^25^. Furthermore, in cases of E-cigarette or Vaping-Associated Lung Injury (EVALI) study, 65% of patients exhibited proteinuria and 20% had microscopic hematuria at hospital admission, with some young patients developing AKI as part of multi-organ failure. Notably, both e-cig users and conventional smokers presented significantly elevated blood cadmium levels compared to non-smokers, and low cadmium concentrations are recognized as a risk factor for CKD^22^.

The assessment of the long-term effects of e-cigarettes is challenging due to their relatively recent widespread adoption. The rapid evolution of devices, the variation in product characteristics such as flavorings, heavy metal content, and nicotine concentration, as well as different user behaviors, complicate data comparison^22^. Moreover, many studies are susceptible to confounding because a large proportion of adult e-cig users—between 40 and 60%—also smoke conventional cigarettes (dual use). The lack of a standard definition for the level of e-cig exposure and the wide variability in inhalation patterns (number of puffs, volume, and duration) further impedes establishing clear dose-response relationships^22^.

Although definitive conclusions on the harm of e-cigarette use to kidneys cannot be currently drawn, the available data point to potential risks to kidney health. The mechanisms underlying the adverse effects are not yet fully elucidated but may involve pathways related to inflammation, oxidative stress, and dysfunction of endothelial, podocyte, and tubular cells^22^.

Given the uncertainties and possible risks of smoking to kidney health, it is imperative that nephrologists actively address the smoking habits of their patients and promote cessation of both conventional cigarettes and e-cigarettes to help prevent CKD progression and improve overall health outcomes.

## Conclusion

This systematic review provides robust evidence that the use of vapers is associated with an increased risk of AKI and CKD, particularly in individuals using high-nicotine devices, compared to conventional tobacco users and non-nicotine users. Research indicates that chronic nicotine exposure from e-cigarettes may exacerbate kidney damage through various mechanisms.

The findings suggest that the renal effects of vaping may be similar to those of conventional cigarettes, highlighting the importance of addressing the risks of vaping in public health initiatives. Future research should focus on delineating the specific mechanisms by which vaping contributes to renal dysfunction and exploring potential strategies to mitigate these risks.

## Data Availability

The dataset supporting the results of this study is not publicly available.
