# Supplementary material for: Effects of vaping on kidney function: a systematic review on acute kidney injury and chronic kidney disease
Source: J Bras Nefrol. 2025 Oct 13;47(4):e20240216. doi: 10.1590/2175-8239-JBN-2024-0216en (PMC12517560; doi:10.1590/2175-8239-JBN-2024-0216en)
Supplement: Translation

# Translation: Efeitos do uso de cigarros eletrônicos na função renal: uma revisão sistemática sobre injúria renal aguda e crônica

**DOI:** 10.1590/2175-8239-JBN-2024-0216pt

**Authors:** Guilherme Nobre Nogueira, Elizabeth De Francesco Daher

**Affiliations:** 1 Universidade Federal do Ceará, Faculdade de Medicina, Departamento de Medicina Interna, Fortaleza, CE, Brasil.; 2 Universidade Federal do Ceará, Faculdade de Medicina, Departamento de Medicina Interna, Programa de Pós-Graduação em Ciências Médicas, Fortaleza, CE, Brasil.

**Keywords:** Vaping, Nicotina, Injúria Renal Aguda, Insuficiência Renal Crônica, Microangiopatia, Lesão Glomerular, Nefrite Intersticial, Glomerulosclerose Segmentar e Focal

## Abstract

O aumento do uso de cigarros eletrônicos, especialmente entre a população mais jovem, tem levado a um maior escrutínio dos seus efeitos na saúde, particularmente na função renal. Este artigo revisa a literatura atual sobre a associação entre cigarros eletrônicos, exposição à nicotina e insuficiência renal, com foco no desenvolvimento de injúria renal aguda (IRA) e doença renal crônica (DRC). A nicotina e outras substâncias químicas presentes nos e-líquidos podem induzir microangiopatia, levando à vasoconstrição e redução da perfusão renal, contribuindo assim para IRA. A exposição crônica à nicotina também promove uma resposta inflamatória, aumentando o risco de nefrite intersticial. Além disso, os danos glomerulares decorrentes do uso contínuo de cigarros eletrônicos têm sido associados à albuminúria e à progressão da glomeruloesclerose segmentar e focal (GESF), condição que pode levar à DRC. Este artigo destaca a necessidade de estudos adicionais para esclarecer os riscos renais associados ao uso de cigarros eletrônicos e fornece informações para políticas de saúde pública sobre o uso da nicotina.

## Introdução

O aumento do uso de cigarros eletrônicos (*vapers*) tem despertado um interesse crescente em seus potenciais efeitos sobre a saúde. Inicialmente comercializados como uma alternativa mais segura aos cigarros convencionais, os cigarros eletrônicos ganharam grande popularidade, especialmente entre a população mais jovem. Embora os efeitos do *vaping* nos sistemas respiratório e cardiovascular tenham sido amplamente investigados, os impactos em outros sistemas orgânicos, particularmente nos rins, permanecem pouco explorados. Os rins filtram os resíduos da corrente sanguínea, mantêm o equilíbrio eletrolítico e regulam a pressão arterial. Consequentemente, quaisquer efeitos prejudiciais à função renal podem ter implicações graves e duradouras para a saúde^1,2^.

Dada a composição química dos líquidos utilizados em cigarros eletrônicos, como nicotina e outras substâncias potencialmente nocivas, os efeitos sobre a função renal são uma preocupação crescente. Alguns estudos sugerem que a nicotina pode induzir vasoconstrição nos rins, contribuindo para injúria renal aguda (IRA) e doença renal crônica (DRC). À luz dos potenciais danos renais a longo prazo, esta revisão sistemática e metanálise visa investigar a associação entre o uso de cigarros eletrônicos e a função renal, com ênfase nos riscos de IRA e DRC^3,4^.

O objetivo deste artigo é fornecer uma análise abrangente da literatura existente sobre os possíveis impactos do cigarro eletrônico na função renal. Especificamente, este estudo busca comparar os efeitos do *vaping* aos do tabagismo convencional e à não utilização de produtos que contêm nicotina. Ao identificar quaisquer diferenças relacionadas a diferentes tipos de *vapers* e concentrações de nicotina, esta análise pode ter implicações significativas para as políticas de saúde pública e recomendações quanto ao uso da nicotina.

## Método

Esta revisão sistemática seguiu a estrutura PICOT. A população de interesse (P) foi composta por indivíduos sem comprometimento renal pré-existente. A intervenção (I) foi o uso de cigarros eletrônicos (e-cigarros) com diferentes concentrações de nicotina. O comparador (C) consistiu em usuários de cigarros convencionais e não usuários de produtos com nicotina. Os desfechos (O) foram alterações na função renal, incluindo o desenvolvimento de IRA e DRC, bem como medições dos níveis séricos de creatinina e da taxa de filtração glomerular (TFG). Para serem incluídos, os estudos deveriam apresentar um período mínimo de acompanhamento de seis meses.

O artigo teve como objetivo responder à seguinte questão: “Qual é a associação entre o uso de *vaping* (e-cigarros) e a ocorrência de IRA e DRC em adultos, em comparação com usuários de tabaco convencional e não usuários de nicotina, em estudos com acompanhamento mínimo de 6 meses?”

A estratégia de busca empregou termos relevantes nas bases de dados PubMed, Embase e Cochrane Library, como segue: [(“*vapers*” OU “*e-cigarettes*” OU “*electronic cigarettes*” OU “*e-cig*” OU “*vaping*” OU “*vape*” OU “*vaporizer*” OU “*vapor products*”) E (“*renal impairment*” OU “*kidney function*” OU “*renal function*” OU “*acute kidney injury*” OU “AKI” OU “*chronic kidney disease*” OU “CKD” OU “*glomerular filtration rate*” OU “GFR” OU “*creatinine*” OU “*renal failure*” OU “*kidney damage*” OU “*nephropathy*”) E (“*tobacco*” OU “*smoking*” OU “*nicotine*” OU “*non-smokers*” OU “*control group*” OU “*cigarette*” OU “*smoking cessation*” OU “*tobacco smoke*” OU “*tobacco use*” OU “*nicotine addiction*” OU “*smoking habits*” OU “*smoking-related diseases*”)].

Um total de 354 artigos foram inicialmente identificados e, após a remoção de 53 duplicatas, 301 foram triados com base nos títulos e resumos. Vinte e quatro artigos na íntegra foram revisados, 10 não mencionavam um grupo controle, 5 não incluíam o uso do cigarro eletrônico como grupo de intervenção e 4 não apresentavam desfechos de interesse. Por fim, cinco artigos preencheram os critérios de inclusão para esta análise, conforme citado na Figura 1.

**Figura 1:** Diagrama de fluxo PRISMA da triagem e seleção dos estudos.

## Resultados

Os estudos incluídos (Tabela 1) relataram, de forma consistente, uma associação entre o uso de cigarros eletrônicos e a função renal prejudicada. Os usuários de e-cigarros apresentaram níveis elevados de creatinina sérica e um declínio na TFG em comparação aos não usuários de produtos com nicotina. Além disso, o risco de IRA e a progressão da DRC foram significativamente maiores entre os usuários de cigarros eletrônicos em comparação aos não fumantes, embora os efeitos fossem, de certa forma, comparáveis aos observados em fumantes de cigarros convencionais.

**Tabela 1:** Descrição Dos artigos incluíDos

Estudo	N	Intervenção	Grupo controle	Desenho do estudo	Tempo de vaping	Acompanhamento	Sexo feminino	Idade média
Poudel 2024	270	Fumantes de tabaco e usuários de cigarros eletrônicos	Não fumantes	Observacional	3,5 ± 2,2 anos	Não especificado	174/270	21,2 ± 2,3
Lyytinen 2023	22	30 tragadas de aerossol de CE com nicotina	Não fumantes	Randomizado	Não especificado	Período de washout de 1 semana	15/22	27 ± 7
Sakamaki-Ching 2020	53	Cigarro eletrônico e cigarro comum	Não fumantes	Transversal	Não especificado	Não especificado	33/53	Não especificado
Bragina 2019	270	Uso de tabaco e cigarro eletrônico	Não fumantes	Observacional	3,5 ± 2,2 anos	Não especificado	174/270	21,2 ± 2,3
Daisy 2024	1315	Uso exclusivo de cigarro eletrônico com e sem nicotina	Não fumantes	Transversal	Não especificado	Não especificado	49,3%	14,9 (mediana)

Entre os cinco estudos incluídos^5–9^, um grande estudo de coorte (Daisy, 2024) relatou que indivíduos que utilizavam cigarros eletrônicos com altas concentrações de nicotina apresentaram um risco significativamente maior de disfunção renal em comparação àqueles que utilizavam concentrações mais baixas. Outro estudo destacou que o uso prolongado de cigarros eletrônicos pode agravar o comprometimento renal devido à exposição contínua a substâncias químicas nocivas encontradas nos líquidos dos cigarros eletrônicos, incluindo metais pesados e outros compostos tóxicos.

No estudo de Daisy (2024), usuários de cigarros eletrônicos contendo nicotina apresentaram níveis plasmáticos mais elevados de cotinina (61,3 ng/mg de creatinina) e 3-HC (118,1 ng/mg de creatinina) em comparação com não usuários (cotinina 0,4 ng/mg de creatinina, 3-HC 0,8 ng/mg de creatinina), indicando maior exposição à nicotina e danos potenciais^5^.

Lyytinen (2023) demonstrou que o aerossol do cigarro eletrônico com nicotina aumentou a formação de trombos plaquetários e a pressão arterial sistólica em +9,25 mmHg. Por outro lado, o aerossol do cigarro eletrônico sem nicotina não apresentou efeitos significativos na trombogênese ou na função microvascular, indicando os efeitos nocivos associados à exposição à nicotina^6^.

Sakamaki-Ching (2020) constatou que os usuários de cigarros eletrônicos apresentaram níveis elevados de marcadores de estresse oxidativo, incluindo 8-OHdG (442,8 ± 300,7 ng/mg) e 8-Isoprostano (750,8 ± 433 pg/mg), em comparação aos não fumantes. Além disso, seus níveis de metalotioneína e zinco foram significativamente mais elevados, sugerindo um aumento da exposição a metais associada a danos oxidativos ao DNA^7^.

Em Poudel (2024), usuários de cigarros eletrônicos apresentaram níveis significativamente mais elevados de albuminúria (252,7 ± 209,1 mg/dia) em comparação com fumantes de tabaco (106,0 ± 76,5 mg/dia) e não fumantes (29,8 ± 31,2 mg/dia), sugerindo maior disfunção renal. Da mesma forma, seu índice de amplificação (Alx) vascular foi elevado (–6,9 ± 11,4%) em relação aos fumantes de tabaco (–12,9 ± 13,7%) e aos não fumantes (–22,0 ± 10,6%)^8^.

Bragina (2019) apresentou achados semelhantes aos de Poudel, com usuários de cigarros eletrônicos exibindo maior albuminúria e disfunção vascular do que fumantes de tabaco e não fumantes, em consonância com ambos os estudos^9^.

Poudel (2024) e Bragina (2019) avaliaram tanto a albuminúria quanto o índice de amplificação (Alx) vascular. Em ambos os estudos, os usuários de cigarros eletrônicos apresentaram os níveis mais elevados de albuminúria e Alx, indicando pior disfunção renal e vascular em comparação com fumantes de tabaco e não fumantes. Os fumantes de tabaco apresentaram resultados intermediários, com albuminúria e Alx elevados em comparação com os não fumantes, mas inferiores aos de usuários de cigarros eletrônicos. Os não fumantes apresentaram os níveis mais baixos em ambos os desfechos^8,9^.

Lyytinen (2023) e Sakamaki-Ching (2020) observaram um aumento de marcadores de danos potenciais (estresse oxidativo, formação de trombos plaquetários) nos usuários de cigarros eletrônicos. Lyytinen (2023) constatou que o aerossol de cigarros eletrônicos contendo nicotina causava um aumento na formação de trombos plaquetários e prejudicava a dilatação microvascular em comparação com o aerossol livre de nicotina. Sakamaki-Ching (2020) identificou um aumento dos marcadores de estresse oxidativo (8-OHdG e 8-Isoprostano) em usuários de cigarros eletrônicos, correlacionado com maior exposição a metais (especialmente zinco) e dano oxidativo ao DNA. Em ambos os estudos, a nicotina nos e-cigarros foi um fator determinante para desfechos adversos, como aumento da trombogênese e estresse oxidativo^6,7^. Os usuários de cigarros eletrônicos contendo nicotina, em ambos os estudos, apresentaram níveis significativamente elevados de cotinina, um marcador de exposição à nicotina.

Daisy (2024) demonstrou uma relação dose-resposta entre a frequência do uso de cigarros eletrônicos e os níveis de cotinina/3-HC, com níveis mais elevados em usuários frequentes. Sakamaki-Ching (2020) identificou uma correlação positiva entre os níveis de cotinina e as concentrações de metais, que foi associada ao dano oxidativo, reforçando a ligação entre o uso de e-cigarros e a injúria renal^5,7^.

Tanto Sakamaki-Ching (2020) quanto Lyytinen (2023) relataram desfechos relacionados ao estresse oxidativo: Sakamaki-Ching (2020) identificou níveis elevados de marcadores de estresse oxidativo, como 8-OHdG, em usuários de cigarros eletrônicos, associados à exposição a metais. Lyytinen (2023) destacou o aumento do dano oxidativo por meio da função microvascular prejudicada após a exposição à nicotina^6,7^.

Todos os estudos observaram níveis elevados de marcadores (por exemplo, albuminúria, cotinina, estresse oxidativo ou trombogenicidade) em usuários de cigarros eletrônicos em comparação aos não usuários. Em todos eles, a nicotina foi identificada como um fator-chave que contribui para desfechos negativos, incluindo aumento do estresse oxidativo, função vascular prejudicada e maior formação de trombos^5,9^.

Essas semelhanças entre os estudos ressaltam os efeitos adversos do uso de e-cigarros - especialmente quando há presença de nicotina - sobre a saúde vascular, o estresse oxidativo e a função renal, representados na Figura 2.

**Figura 2:** Efeitos potenciais e mecanismos do *vaping* na saúde renal.

## Discussão

Estudos recentes confirmaram que a nicotina, independentemente do método de administração, representa um risco significativo para a saúde renal. Por exemplo, Patel et al.^10^ observaram que a exposição à nicotina causa vasoconstrição e estresse oxidativo nos tecidos renais, aumentando a probabilidade de IRA e DRC em usuários crônicos. Esses achados estão alinhados com os resultados desta revisão, indicando que os usuários de cigarros eletrônicos apresentam risco de danos renais comparável ao de fumantes de cigarros convencionais.

Além da nicotina, diversos compostos encontrados no vapor produzido pelos cigarros eletrônicos, como o propilenoglicol, o glicerol e os agentes aromatizantes, podem contribuir para a disfunção renal. Zhang et al.^11^ exploraram como os produtos da decomposição desses compostos podem levar à disfunção endotelial na vasculatura renal, o que poderia exacerbar ou iniciar a injúria renal. Esses achados apoiam a hipótese de que, embora o uso de cigarros eletrônicos possa reduzir certos riscos associados aos cigarros tradicionais, ele introduz novos riscos à função renal.

Em contrapartida, alguns estudos sugerem que o tipo de dispositivo de vaporização e a concentração de nicotina podem alterar os riscos para a saúde renal. Por exemplo, indivíduos que utilizam cigarros eletrônicos com baixo teor de nicotina ou aqueles projetados para liberar nicotina de forma mais lenta parecem apresentar um risco reduzido de comprometimento renal em comparação aos que utilizam dispositivos com alto teor de nicotina. São necessários estudos adicionais para confirmar essas observações e compreender os mecanismos pelos quais diferentes produtos de vaporização afetam a função renal.

Fumantes atuais e ex-fumantes apresentam um risco significativamente maior de hospitalização por IRA em comparação com indivíduos que nunca fumaram. Esse risco diminui com o tempo após a cessação do tabagismo, mas pode persistir por até 30 anos^12^. A exposição ao vapor do cigarro eletrônico, especialmente quando associada a uma dieta rica em gordura, aumenta o estresse oxidativo, os danos ao DNA e as respostas inflamatórias nos rins, acelerando o desenvolvimento de patologias renais^13^. Pacientes com histórico de IRA apresentam um risco aumentado de desenvolver DRC e doença renal em estágio terminal (DRET). Esse risco é maior em comparação àqueles sem histórico de IRA, e a gravidade da IRA está associada a desfechos mais desfavoráveis^14^.

Fumantes com IRA apresentam níveis significativamente mais baixos de vitamina D em comparação com não fumantes. Essa deficiência pode exacerbar complicações inflamatórias e piorar os desfechos renais. A vitamina D é conhecida por desempenhar um papel crucial na modulação das respostas imunológicas e na redução da inflamação, e sua deficiência pode levar ao aumento da suscetibilidade a danos renais e à progressão da doença renal^15^.

Estudos indicam que tanto o tabagismo convencional quanto o *vaping* estão associados a um risco aumentado de injúria renal aguda e crônica. Fumantes de cigarros convencionais apresentam um risco elevado de IRA, que pode persistir por décadas após a cessação do tabagismo. Além disso, fumantes com IRA frequentemente apresentam deficiência de vitamina D, o que pode exacerbar ainda mais os desfechos renais.

A inflamação crônica induzida pela nicotina também é um fator significativo no desenvolvimento da nefrite intersticial. Embora não existam muitos estudos que se concentrem especificamente nesta condição em usuários de cigarros eletrônicos, a literatura existente sobre o tabagismo tradicional sugere que a inflamação renal mediada pela nicotina, agravada por respostas imunológicas alteradas, pode levar à nefrite intersticial. O mesmo processo pode ser extrapolado para usuários de cigarros eletrônicos, considerando a semelhança dos danos causados pela exposição crônica à nicotina^4,8,10^.

Um aspecto relevante é a associação entre nicotina e disfunção glomerular. Os níveis elevados de albuminúria observados em usuários de cigarros eletrônicos, conforme observado nos estudos analisados, são indicativos de dano glomerular, que pode evoluir para GESF. Além disso, a exposição contínua a metais pesados presentes nos líquidos dos cigarros eletrônicos pode intensificar o processo de esclerose glomerular, sugerindo que usuários de dispositivos com alto teor de nicotina podem apresentar um risco elevado de desenvolver GESF^6,10^.

Em contrapartida, os não usuários de nicotina geralmente apresentam melhores desfechos de saúde renal, indicando que os danos oxidativos e inflamatórios relacionados à nicotina estão associados à ocorrência de IRA e DRC. Isso destaca a necessidade de estudos adicionais para esclarecer os impactos renais de longo prazo do uso de cigarros eletrônicos em comparação ao tabagismo tradicional e ao não uso de produtos de tabaco.

Além da nicotina, os componentes não-nicotínicos dos aerossóis dos cigarros eletrônicos—tais como ácidos graxos livres (AGL) e outros agentes lipofílicos—podem desempenhar um papel na injúria renal. Os AGL demonstraram induzir lipotoxicidade nas células endoteliais renais e nos podócitos, promovendo disfunção mitocondrial, estresse do retículo endoplasmático e geração de espécies reativas de oxigênio (ERO)^16,17^. Esses mecanismos podem comprometer a integridade glomerular e acelerar a progressão da DRC. Estudos *in vitro* recentes sugerem que os aromatizantes químicos utilizados nos e-líquidos também podem aumentar os níveis de AGL e alterar o metabolismo lipídico nas células expostas^18^, o que poderia potencializar a nefrotoxicidade independentemente da nicotina. A interação entre a peroxidação lipídica, a inflamação e as respostas ao estresse celular pode, portanto, fornecer uma via explicativa adicional para os danos renais observados em usuários de cigarros eletrônicos.

O uso de cigarros eletrônicos também pode afetar outros órgãos do trato geniturinário, particularmente a bexiga. Estudos recentes levantaram preocupações sobre o potencial carcinogênico do uso de e-cigarros, com ênfase especial em neoplasias malignas uroteliais. A excreção urinária de metabólitos derivados de nitrosaminas e hidrocarbonetos policíclicos aromáticos, compostos encontrados tanto nos cigarros tradicionais quanto nos eletrônicos, pode resultar na exposição direta do epitélio da bexiga a carcinógenos. Por exemplo, Lee et al.^19^ identificaram níveis elevados de N-nitrosaminas na urina de usuários de cigarros eletrônicos, compostos reconhecidamente carcinogênicos para a bexiga. Além disso, Werley et al.^20^ relataram danos ao DNA e estresse oxidativo em células uroteliais expostas a extratos de aerossóis de cigarros eletrônicos. Embora os dados epidemiológicos ainda sejam limitados, os achados pré-clínicos sustentam uma ligação mecanicista entre o uso crônico de cigarros eletrônicos e o aumento do risco de câncer de bexiga, particularmente em indivíduos com outros fatores de risco, como sexo masculino, exposição ocupacional e predisposição genética^19–21^. Essas preocupações emergentes ressaltam a necessidade de estudos prospectivos para avaliar o impacto oncológico de longo prazo do uso de cigarros eletrônicos no sistema geniturinário.

O tabagismo convencional é um fator de risco bem estabelecido para o desenvolvimento de DRC, para a aceleração de DRC pré-existente e para a falência do aloenxerto renal^22,23^. É importante ressaltar que a cessação do tabagismo tem se mostrado eficaz na redução desse risco^23^. Nos últimos anos, entretanto, houve um aumento dramático no uso de cigarros eletrônicos (e-cigs), também conhecidos como *vapes*. Apesar de sua crescente popularidade, os efeitos do uso de e-cigs na saúde renal em comparação com os cigarros convencionais permanecem desconhecidos^22^.

Estudos experimentais em animais sugerem que os componentes e aerossóis dos cigarros eletrônicos podem causar danos estruturais e funcionais aos rins. Por exemplo, camundongos expostos, durante seis meses, ao vapor de cigarros eletrônicos contendo nicotina apresentaram uma redução significativa na taxa de filtração glomerular, acompanhada de aumento da fibrose renal^24^. Outras pesquisas em ratos apontam o próprio líquido de recarga dos cigarros eletrônicos, e não a nicotina, como a principal causa de danos estruturais aos rins^22^. Além disso, em camundongos obesos e diabéticos, a exposição prolongada a aerossóis de cigarros eletrônicos contendo nicotina induziu alterações significativas na expressão gênica relacionada à progressão da DRC. Complementando esses achados, estudos *in vitro* revelaram que a vanilina, um agente aromatizante comum nos líquidos de cigarros eletrônicos, exibe efeitos citotóxicos nas células tubulares renais humanas, prejudicando a viabilidade mitocondrial e as vias autofágicas. Adicionalmente, a exposição materna ao vapor de cigarros eletrônicos durante a gestação e a lactação, em modelos murinos, resultou em descendentes com densidade glomerular reduzida e aumento da albuminúria^22^.

Apesar desses achados experimentais, as evidências de estudos em humanos permanecem limitadas e derivam, principalmente, de um pequeno número de investigações transversais^22^. Um estudo envolvendo adolescentes e adultos jovens com DRC de início pediátrico observou uma tendência ao aumento da proteinúria entre usuários de cigarros eletrônicos, embora sem significância estatística. Outro estudo transversal, realizado em indivíduos jovens saudáveis, relatou uma prevalência mais elevada de albuminúria entre usuários de cigarros eletrônicos em comparação com fumantes de cigarros tradicionais e não fumantes^25^. Além disso, nos casos relatados no estudo *E-cigarette or Vaping-Associated Lung Injury* (EVALI), 65% dos pacientes apresentaram proteinúria e 20% apresentaram hematúria microscópica na admissão hospitalar, com alguns pacientes jovens desenvolvendo IRA como parte de uma falência múltipla de órgãos. Notavelmente, tanto os usuários de cigarros eletrônicos quanto os fumantes convencionais apresentaram níveis significativamente elevados de cádmio no sangue em comparação com os não fumantes, e concentrações mesmo baixas de cádmio são reconhecidas como um fator de risco para DRC^22^.

A avaliação dos efeitos a longo prazo dos cigarros eletrônicos é desafiadora devido à sua adoção relativamente recente em larga escala. A rápida evolução dos dispositivos, a variação nas características dos produtos, como aromatizantes, teor de metais pesados e concentração de nicotina, bem como os diferentes comportamentos dos usuários, tornam complexa a comparação dos dados^22^. Além disso, muitos estudos são suscetíveis a fatores de confusão, pois uma grande proporção dos usuários adultos de cigarros eletrônicos—entre 40% e 60%—também fuma cigarros convencionais (uso duplo). A ausência de uma definição padrão para o nível de exposição ao cigarro eletrônico e a ampla variabilidade nos padrões de inalação (número de tragadas, volume e duração) dificultam ainda mais o estabelecimento de relações dose-resposta claras^22^.

Embora não seja possível, atualmente, estabelecer conclusões definitivas sobre os danos causados pelo uso de cigarros eletrônicos aos rins, os dados disponíveis apontam para riscos potenciais à saúde renal. Os mecanismos subjacentes aos efeitos adversos ainda não foram totalmente elucidados, mas podem envolver vias relacionadas à inflamação, ao estresse oxidativo e à disfunção das células endoteliais, podocitárias e tubulares^22^.

Dadas as incertezas e os possíveis riscos do tabagismo para a saúde renal, é imperativo que os nefrologistas abordem ativamente os hábitos tabágicos de seus pacientes e promovam a cessação do uso tanto de cigarros convencionais quanto de cigarros eletrônicos, a fim de contribuir para a prevenção da progressão da DRC e melhorar os desfechos gerais de saúde.

## Conclusão

Esta revisão sistemática fornece evidências robustas de que o uso de cigarros eletrônicos está associado a um risco aumentado de IRA e DRC, particularmente em indivíduos que utilizam dispositivos com alto teor de nicotina, em comparação com usuários de tabaco convencional e não usuários de nicotina. Pesquisas indicam que a exposição crônica à nicotina dos e-cigarros pode agravar o dano renal por meio de diversos mecanismos.

Os achados sugerem que os efeitos renais do uso de cigarros eletrônicos podem ser semelhantes aos observados com o tabagismo convencional, destacando a importância de abordar os riscos do *vaping* em iniciativas de saúde pública. Pesquisas futuras devem se concentrar em delinear os mecanismos específicos pelos quais o uso de cigarros eletrônicos contribui para a disfunção renal e explorar potenciais estratégias para mitigar esses riscos.

## Data Availability

O conjunto de dados que sustenta os resultados deste estudo não está disponível ao público.
